# Diagnostic delay of oral squamous cell carcinoma and the fear of diagnosis: A scoping review

**DOI:** 10.3389/fpsyg.2022.1009080

**Published:** 2022-11-03

**Authors:** Rodolfo Mauceri, Monica Bazzano, Martina Coppini, Pietro Tozzo, Vera Panzarella, Giuseppina Campisi

**Affiliations:** ^1^Department of Surgical, Oncological and Oral Sciences (Di.Chir.On.S.), University of Palermo, Palermo, Italy; ^2^U.O.C. of Stomatology, A.O.O.R., Villa Sofia-Cervello of Palermo, Palermo, Italy; ^3^Unit of Oral Medicine and Dentistry for Fragile Patients, Department of Rehabilitation, Fragility and Continuity of Care, University Hospital Palermo, Palermo, Italy

**Keywords:** oral squamous cell carcinoma, patient delay, professional delay, treatment delay, fear, cancerphobia

## Abstract

The mortality rate of patients affected with oral squamous cell carcinoma (OSCC) has been stable in recent decades due to several factors, especially diagnostic delay, which is often associated with a late stage diagnosis and poor prognosis. The aims of this paper were to: analyze diagnostic delay in OSCC and to discuss the various psychological factors of patients with OSCC, with particular attention to the patient’s fear of receiving news regarding their health; and the professional dynamics related to the decision-making processes in cases of suspected OSCC. A preliminary review of literature focusing on OSCC diagnostic delay was performed. Seven articles were included with the diagnostic delay ranging from 45  days to approximately 6  months. Patients’ fears and, to a lesser degree, the concerns of dentists, were found to be still poorly investigated. On the basis of the authors’ professional experience, the development of oral lesions of unknown origin may generate different behaviors in the decision-making processes by patients and clinicians, and fear may play a key role in the distinct steps of this process. It is crucial to increase awareness and inform patients about the onset of OSCC, and contemporaneously encourage experimental studies on patients’ fear and professional behaviors with respect to communication regarding OSCC.

## Introduction

Oral cancer is one of the most common malignancies in the world, with global estimates indicating that there are approximately 354,864 new cases per year. Of these, approximately 90% of cases are OSCC (World Health Organization – Global Cancer Observatory-GLOBOCAN, 2020). According to the GLOBOCAN 2020 data, OSCC ranks 16th by incidence (with 377,713 new cases/year) and annual mortality (177,757 deaths). The GLOBOCAN 2020 data referring to Italy documented 4,037 new cases, 1,585 deaths and a 5-year prevalence of 12,519 OSCC patients (World Health Organization – Global Cancer Observatory-GLOBOCAN, 2020). OSCC is a neoplasm currently with a poor prognosis, and mortality rates are very high in most countries. The mortality rate of patients with OSCC in Italy is very high and, unfortunately, this rate has remained stable for more than 20 years (World Health Organization – Global Cancer Observatory-GLOBOCAN, 2020).

Survival data is strongly influenced by the timing of diagnosis: more than 50% of patients with OSCC are diagnosed at an advanced stage, and the 5-year survival rate of these patients is less than 50%, a fact which is mainly due to diagnostic delay (La Vecchia et al., 2004; Goy et al., 2009; Gatta et al., 2015; Gigliotti et al., 2019). Indeed, an early diagnosis of OSCC plays a crucial role in improving a patient’s prognosis, as a five-year survival period is more than 90% if diagnosed early, falling to 5–20% for a late diagnosis (stage III and IV; Ragin et al., 2007; Gatta et al., 2015).

The term *diagnostic delay* generally refers to the time elapsing between the moment when the patient recognizes the first sign or symptoms of illness and the time when a definitive diagnosis is made, following a specialist examination (Scott et al., 2006). Diagnostic delay consists of an *appraisal interval* and a *help-seeking interval*, both of which are attributed to the patient (also known as *patient delay*), whilst *professional delay* is attributed to the health professional. The *appraisal interval* refers to the period of time from the moment the patient becomes aware of the signs and/or symptoms of illness until the moment they decide to consult a doctor. The *help-seeking interval* refers to the period of time from the moment of perception of the need for further diagnostic investigation to the initial consultation (Scott et al., 2006). *Professional delay* refers to the period of time which elapses from an initial consultation, usually at primary care facilities, until a definitive histopathological diagnosis is made as a result of one or more specialist consultations and referral to a clinic specializing in the treatment of head and neck cancers (van der Waal et al., 2011).

Of the known factors related to diagnostic delay, the literature suggests that one of the main causes of delay attributed to the patient is a lack of knowledge regarding OSCC and related risk factors (Warnakulasuriya et al., 1999; Al-Kaabi et al., 2016). In addition, the absence of the pathognomonic signs or symptoms of OSCC often leads patients to incorrectly attribute these signs or symptoms to infections or dental problems (Rogers et al., 2011; van der Waal et al., 2011; Leuci et al., 2017). As regards the factors contributing to professional delay, a decisive role can be played by the lack of information, awareness and training of healthcare professionals (Silverman et al., 2010; Kebabcıoğlu and Pekiner, 2018; Grafton-Clarke et al., 2019). To date, there have been few, if any, studies in the scientific literature analyzing *fear* as a psychological variable, which seems to determine the diagnostic delay of patients and dentists (Panzarella et al., 2014). Therefore, studies relating to diagnostic delay in the literature in this scoping review and fear-related OSCC will be analyzed in order to hypothesize if and to what extent fear intervenes in patients’ and dentists’ choices regarding the diagnostic delay of OSCC.

## Materials and methods

A review of the recent literature was conducted, focusing on the diagnostic delay in patients with OSCC (as described below). This initial search revealed 177 publications in the 2011–2021 period, and 7 studies in English, from which pertinent information was obtained, were included in the review. Where available, these studies were used as the main source of information. This revision revealed a lack of robust clinical trials and specific information, making it problematic to develop concepts and recommendations, which were based solely on scientific evidence.

### Eligibility criteria

The inclusion criteria in this review were: (I) patients affected by OSCC; (II) a minimum number of at least 25 patients in the study; (III) data defining the mean of patient delay and/or professional delay or a total diagnostic delay (in days or months); and (IV) studies which had been published in the previous 10 years. The exclusion criteria were: (I) studies including patients with other types of cancer; (II) studies including less than 25 patients affected by OSCC; (III) research involving animals and *in vitro* studies; (IV) systematic and narrative reviews, expert opinions; and (V) studies written in languages other than English. Research results not satisfying the inclusion criteria were excluded during data collection.

### Information sources

Studies were identified by an electronic search of scientific articles from different biomedical databases (e.g., PubMed, Ovide/MEDLINE, Web of Knowledge, Embase) and by scanning reference lists of articles; the choice of language was unlimited. This literature review included scientific articles for the 1 November 2011 to 1 November 2021 period.

### Search

The following search terms were used separately and in combination: oral squamous cell carcinoma, OSCC, oral tongue squamous cell carcinoma; squamous cell carcinoma of the mouth, delayed diagnosis, fear, using medical subject headings and free text.

### Study selection

The eligibility assessment was performed independently by two reviewers (M.B. and R.M.) and any discrepancies between reviewers were resolved by consensus. The studies were initially selected by applying the inclusion and exclusion criteria of the study and abstract titles. Duplicate papers were deleted, after which there followed further scrutiny in order to assess their eligibility.

## Results

**Figure 1:**
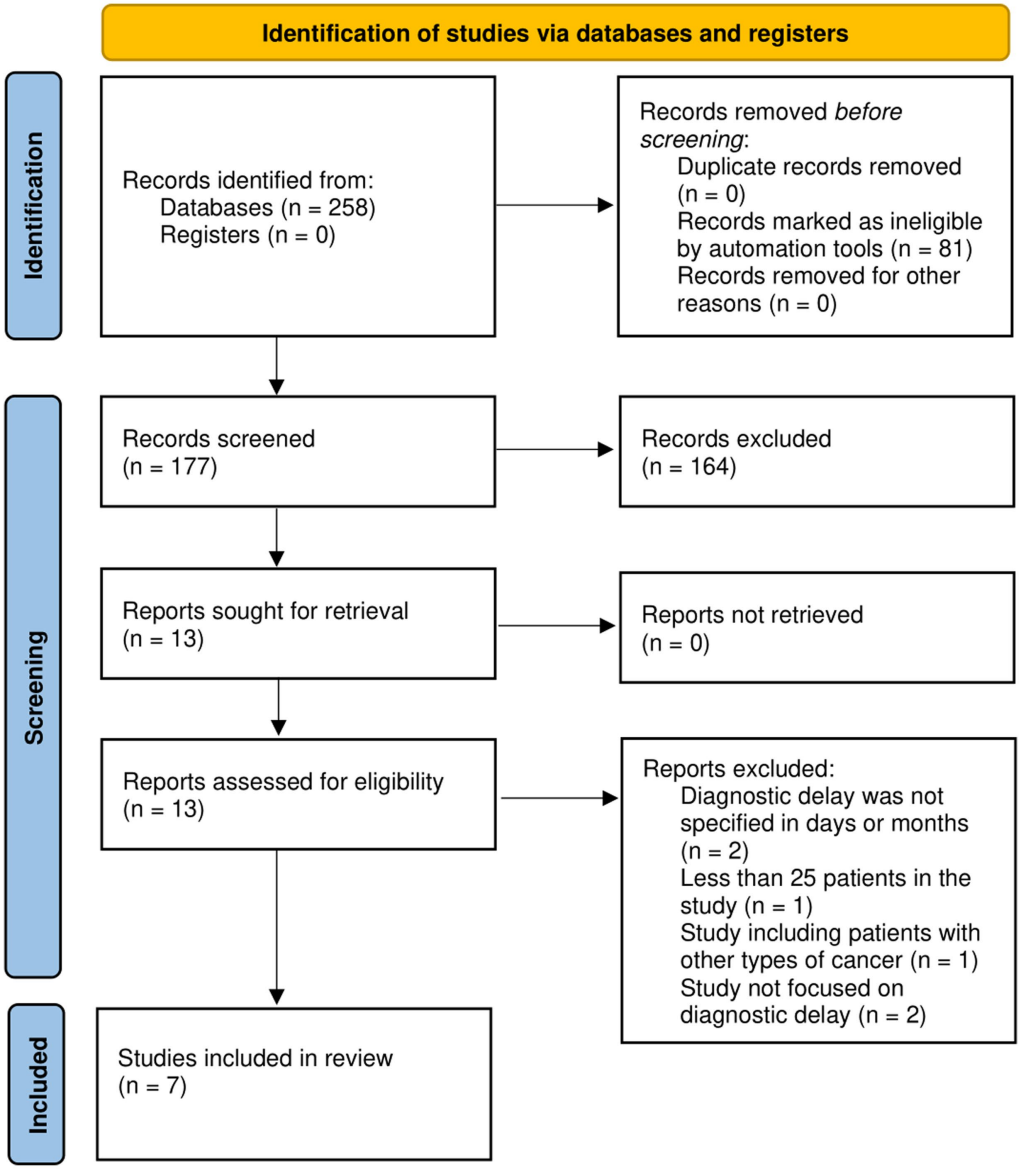
PRISMA flow diagram for systematic reviews.

Searching through the various databases yielded 177 articles whilst an additional record search identified 2 articles. Having applied the eligibility assessment, 16 full-text studies were included. Of the selected manuscripts, an additional 9 were excluded during the full-text analysis, thereby leaving a total 7 of articles included in this review (Figure 1; Seoane-Romero et al., 2012; Joshi et al., 2014; Baishya et al., 2015; Kerdpon et al., 2018; Marella et al., 2018; Rutkowska et al., 2020; Thomas et al., 2021). The evaluated information of the selected studies was: the sample size, patient, country, professional and total delay. Due to the heterogeneity of the included studies, a scoping review was performed.

Several studies in the past decade have evaluated the diagnostic delay in OSCC patients by analysing patient and/or professional delay (Table 1). Of the studies included, the majority were conducted in Europe (1 in Italy, 1 in Spain and 1 in Poland), south-east Asia (2 in India and 1 in Thailand), and 1 in Australia. The range of diagnostic delay ranged from 45 days to 7.4 months with most studies displaying an average diagnostic delay of approximately 5 months (except Seoane-Romero et al. with 45 days; Seoane-Romero et al., 2012; Joshi et al., 2014; Baishya et al., 2015; Kerdpon et al., 2018; Marella et al., 2018; Rutkowska et al., 2020; Thomas et al., 2021). Regarding study design, there were 3 prospective studies (Joshi et al., 2014; Baishya et al., 2015; Kerdpon et al., 2018), three retrospective studies (Marella et al., 2018; Rutkowska et al., 2020; Thomas et al., 2021) and 1 cohort study (Seoane-Romero et al., 2012). A total of 1,038 patients were enrolled in the seven studies included and the majority were males [610/907; 67%; except Baishya et al. (2015)] (Seoane-Romero et al., 2012; Joshi et al., 2014; Kerdpon et al., 2018; Marella et al., 2018; Rutkowska et al., 2020; Thomas et al., 2021). The patients’ mean age ranged from 48 to 68.5 years (Seoane-Romero et al., 2012; Joshi et al., 2014; Baishya et al., 2015; Kerdpon et al., 2018; Marella et al., 2018; Rutkowska et al., 2020; Thomas et al., 2021).

**Table 1:** Summary of studies from 2011 to 2021 regarding the diagnostic delay in OSCC.

Author (year)	Country	Design	Sample size	Male/Female patient	Mean age	Early TNM Stage (I and II)	Late TNM Stage (I and II)	Patient delay (days or month)	Professional delay (days or month)	Total delay (days or month)
Seoane-Romero et al. (2012)	Spain	Cohort study	88	58–30	60.3	40	48	n.d.	n.d.	45 days
Joshi et al. (2014)	India	Prospective study	201	172–29	48	0	201	2.75 month	3.34 month	6.09 month
Baishya et al. (2015)	India	Prospective study	57 Tongue 74 Mouth	131*	55.4	n.d.	n.d.	90 days 60 days	n.d.	n.d.
Kerdpon et al. (2018)	Thailand	Prospective study	154	98–56	61.4	n.d.	n.d.	100 days	67.7 days	161.1 days
Marella et al. (2018)	Italia	Retrospective study	59	26–33	68.5	n.d.	n.d.	112 days	40 days	152 days
Rutkowska et al. (2020)	Poland	Retrospective study	305	193–112	60.7	81	224	7.4 month	n.d.	n.d.
Thomas et al. (2021)	Australia	Retrospective study	100	63–37	63.4	68	32	n.d.	n.d.	4 month

* Gender data not defined. Unavailable data are labelled by “n.d.” (not defined).

Based on the available information of the tumour site on 879 patients (except Marella et al. and Thomas et al.), the tongue was affected by OSCC on 209 patients and the mouth by 516 lesions (Seoane-Romero et al., 2012; Joshi et al., 2014; Baishya et al., 2015; Rutkowska et al., 2020). Kerdpon et al. described 154 patients affected by OSCC without, however, defining their location (Kerdpon et al., 2018). Only four studies described the TNM stage of the patients: 189 patients (189/694; 27.2%) were in the early stages of advancement (stage I and II) and 504 patients (505/694, 72.8%) were in the late stages of OSCC (stage III and IV; Seoane-Romero et al., 2012; Joshi et al., 2014; Rutkowska et al., 2020; Thomas et al., 2021).

None of the studies analyzed in detail the differences between the appraisal interval and/or help-seeking interval, thus merely providing data relating to the sum of these two latter items (i.e., patient delay). The lowest intervention reaction time between signs/symptoms and definitive diagnosis were demonstrated by Seoane-Romero et al.: the authors demonstrated that the median for the interval between the first sign/symptom and pathological diagnosis was 45 days. In addition, most patients (54.5%) suffered no delayed diagnosis. This, therefore, led to the diagnosis of many cases of OSCC in its early stages (I–II; Seoane-Romero et al., 2012).

Joshi et al. evaluated diagnostic delay in India, demonstrating a patient-related delay of 2.75 months, associated with a professional delay of 3.34 months (total 6.09 months) in a cohort of 201 OSCC patients. A second study by Baishya et al. evaluated only patient delay, it revealed a delay of 90 days in patients with OSCC only of the tongue and 60 days in patients with OSCC in the other mucosa sites in the mouth. Another study originating in South-East Asia discussed similar delay periods, with an average patient delay of 100 days and a professional delay of 67.7 (Kerdpon et al., 2018). In a study regarding Italian patients, Marella et al. (2018) confirmed a diagnostic delay of 152 days, comprising a patient delay of 112 days and professional delay of 40 days. And, in a recent study by Rutkowska et al. (2020), the mean patient delay was prolonged, being evaluated at over 7 months. The final study to be mentioned in this paper regarded Australian patients with OSCC, in which only the total diagnostic delay time was evaluated, being defined as approximately 4 months (Thomas et al., 2021). None of the included papers described any results regarding the role of fear in the diagnostic delay of OSCC.

## Discussion

Due to the paucity of information in the studies reviewed in this paper, it can be stated that there is a wide range in the total delay in OSCC patients of between 45 days and approximately 6 months. Regarding sex, the prevalence of males in the sample (67%) supports the data from the World Health Organization (WHO) (World Health Organization – Global Cancer Observatory-GLOBOCAN, 2020). The age of most patients over 60 also concurs with the data from the WHO and scientific literature (Edwards and Aronson, 2000; World Health Organization – Global Cancer Observatory-GLOBOCAN, 2020; da Cunha Lima et al., 2021; Gissi et al., 2021; Panzarella et al., 2021). With only 4 papers describing the TNM stage, most of the patients were in the late stages of OSCC (505/694; 72.8%; Seoane-Romero et al., 2012; Joshi et al., 2014; Rutkowska et al., 2020; Thomas et al., 2021). This datum confirms that more than 50% of patients with OSCC are diagnosed at an advanced stage (La Vecchia et al., 2004; Goy et al., 2009; Gatta et al., 2015; Gigliotti et al., 2019; Fontana et al., 2021).

Of interest, Rutkowska et al. (2020) and Joshi et al. (2014) described a more significant diagnostic delay (7.4 and 6.09 months respectively), having simultaneously enrolled patients with late stage OSCC. These data are in contrast with Thomas et al., who described a total delay of 4 months but more early stages than late stage of OSCC. Remarkably, Seoane-Romero et al. reported a very low diagnostic delay (45 days), which was associated with an equal distribution of early and late stages of OSCC (Seoane-Romero et al., 2012; Thomas et al., 2021). One of the main causes of diagnostic delay can be attributed to the patient’s lack of knowledge regarding OSCC, its pathognomonic signs or symptoms and related risk factors (Warnakulasuriya et al., 1999; Al-Kaabi et al., 2016). However, the latter may mask significant factors relating to the progression of OSCC in the absence of strong evidence regarding patient delay, as obtained from scientific data.

The available scientific literature relating to other types of cancer (e.g., breast cancer) suggests that fear seems to be the main reason why patients decline to participate in cancer screening programmes and periodic check-up appointments. Notwithstanding the numerous studies on this topic, the evidence is still limited (Mohan et al., 2021; Muñoz-Sanz et al., 2021; Taylan et al., 2021; Richter et al., 2022). It is widely agreed by many health professionals that fear is a fundamental emotion in the array of human reactions, which can be understood as a defence mechanism; this, therefore, protects the healthy individual, which in turn is indispensable for survival. The faculty to challenge the notion that the duration of human life is finite can minimise the end-of-life anguish of many human beings (Heidegger, 2008). It can also be stated that many human beings are aware that these two existential phenomena - *duration* and *an end* – are the sum total of a human life. Thus, when faced with a life-threatening situation, a series of reactions can be activated, which serve to protect life from danger, and this includes an extremely stressful situation such as a diagnosis of cancer (Wells, 1997). And it is at this juncture that a patient’s “perception time” of the initial signs and symptoms of a disease requiring investigation comes to the fore.

To date, patient delay can be divided into an *appraisal interval*, that is the moment of the perception of initial signs and symptoms, and a *help-seeking interval*, or the moment of perception of the need for further diagnostic investigation (Coxon et al., 2018). And it is precisely during these periods of perception that the fear of cancer often seems to be expressed indirectly through the fear of pursuing cancer-related investigations.

Such a defence mechanism invariably negates the confirming of a diagnosis, thereby permitting the patient to expunge the fear of falling ill (Pozza et al., 2015). The fear of illness has long existed because it is inherent in human nature. Illness can be regarded as a stressful life event, even more so when it involves an oncological disease, threatening the body from within. It is a truism that going to the doctor for a check-up or a preliminary examination can be such an anxiety-inducing experience for some people that it is preferable to avoid any form of medical care (Baishya et al., 2015). Indeed, this fear of doctors is termed *iatrophobia* in the literature. The most intense fear for those suffering from *iatrophobia* can be said to be the fear of the unknown and of being confronted with potentially dangerous diseases or situations (Chambless and Gracely, 1989). A careful scrutiny of listening to patients’ musings often reveals that, at the very moment of thinking about seeing the dentist, negative thoughts prevail: the patient begins to imagine dire situations or interminably painful and often expensive check-ups (Stefanuto et al., 2014). Such a consequence is not only driven by the fear and avoidance of pain but also the desire to avoid “bad news,” including a diagnosis of cancer. And any discussion regarding the origins of fear must make reference to the following: the physiological/biological, the psychological/personological and environmental factors (Perna et al., 2004). The mere thought of facing a diagnosis of cancer, with all its life (or death) consequences can encourage intrusive thoughts, leading to a “flooding of the mind” (Torta and Caldera, 2008).

The executive functions of the frontal lobe, which processes information and which is involved in decision-making, no longer function efficiently, concurrent with the activation of the limbic system and the amygdala, thereby signaling a state of emergency (Akirav and Maroun, 2007). This reaction can occur when one’s surroundings are not comprehended, when the “thinking brain” (frontal lobe neocortex) would otherwise be able to assign meaning to a given situation (Rosen, 2004). It constitutes an effective neuroanatomical barrier whereby the presence of a strong emotional state prevents new information from being processed rationally (Johansen et al., 2011; Sears et al., 2014; Campese et al., 2016). However, it is to be hoped that the patient will be able to seek assistance with the aim of obtaining psychological support. Thus, control over their emotions and stable mental equilibrium will be reasserted as soon as is possible. Regrettably, this situation often remains beyond the dentist’s control, leading to with patients who present with potentially malignant lesions or OSCC.

Any professional delay attributed to the health professional may be due to various known factors (e.g., a lack of clinical competence, the prescribing of inappropriate diagnostic investigations) but also to more psychological factors (Singh and Schenberg, 2013; Joshi et al., 2014; Wells et al., 2016; Kerdpon et al., 2018; Marella et al., 2018). In spite of the benefits of performing a simple biopsy in the presence of suspicious lesions, oral incisional biopsies appear not to be so common in dental practice. This may be due to the clinician’s perceptions of: the training deficits and the risk of diagnostic error related to the fear of a medicolegal complication, unfamiliarity with the biopsy technique, and the misconception that it is a predominantly specialist procedure (Logan and Goss, 2010; Murgod et al., 2011; da Cunha Lima et al., 2021). Indeed, Rutkowska et al. have highlighted that in their study that the most common causes of professional delay were: the prescription of antibiotics, mouth rinse prescriptions and other pharmacotherapies; the collection of inappropriate bioptic samples and a lack of oncological awareness (Rutkowska et al., 2020).

This tendency and these fears may lead the clinician to refer the patient to other centers or, even worse, to wait for extended periods of time prior to a referral. Thus, the total delay time is increased, impacting the patient’s prognosis (Logan and Goss, 2010; Murgod et al., 2011; da Cunha Lima et al., 2021). It is the considered opinion of the authors of this study that for a dentist to merely utter the “unspeakable” word *cancer* is to inflict psychological pain. Many dentists could fear irreparably damaging their relationship with the patient by communicating such potentially poor results, which could inevitably change the patient’s life. Indeed, the dentist may also fear illness and death, although many would consider it inappropriate to identify with the patient (e.g., reciprocity of pain, possibly excessive empathy), thereby activating defence mechanisms. Thus, the dentist may feel professionally unprepared to deal with the challenges of expressing and managing emotions (Rutkowska et al., 2020; da Cunha Lima et al., 2021). In addition, such fears can often be accompanied by errors of judgment in this delicate phase of dentist-patient interaction of communicating “bad news.” This is probably due to the fact that some dentists lack effective training regarding the communicating of a poor prognosis, and on occasion they could provide too much information during one appointment, some of which may be unnecessary (Rutkowska et al., 2020; Bazzano et al., 2021). Where it is considered necessary to perform diagnostic tests in patients with suspected OSCC, the dentist could avoid the discomfort of negative communication and the perceived anxiety of listening (Rutkowska et al., 2020).

### Limitations of the study

The present study possesses various limitations. Few studies could be included in this review and the referenced studies were rather heterogeneous in nature. The review includes data from a range of countries, ranging from developed to emerging economies. Socioeconomic conditions may be associated with a delayed diagnosis of OSCC and only two studies mentioned in this study investigated the location of patient residence, with three studies mentioning patients’ educational level (Baishya et al., 2015; Kerdpon et al., 2018). Given that the evaluation of patient delay was one aim of this research, these conditions may seriously compromise the time period for patients to be able to physically access medical facilities. Furthermore, it may be the case that professional delay can differ from more developed to emerging economies. Nor has it been established that rural patients receive the same level of treatment as urban patients.

As previously stated, one major limitation in this study is possibly the lack of specific information regarding patient delay (appraisal interval vs. help-seeking interval) and professional delay.

The results of this review are not absolute, due to the absence of robust clinical trial and specific information on the topic. Such a deficit has led to the construction of the notion of fear being based on the expert opinion of the authors of this study, as garnered in public oral oncology centers in recent decades, who believe that future research is necessary and should be based on a strong and competent interdisciplinary approach to the psychological sciences. However, to best of these authors’ knowledge, this is the first paper which has analyzed the role of fear in the diagnostic delay of OSCC.

### Future perspectives

To date there have been limited studies regarding diagnostic delay in the literature, with little or no analysis of the individual moments of dentist-patient interaction. Indeed, the few authors who have undertaken these studies have limited their investigation to describing patient and/or professional delay. Given the difficulty in acquiring precise data and the considerable psychological component of these processes, it is no easy matter to distinguish the appraisal interval from the help-seeking interval by patient. However, there are numerous health campaigns whose aims are to raise awareness and inform patients and dentists about the risk factors of OSCC, self-diagnosis and early diagnosis, thereby encouraging a reduction in patient-related diagnostic delay. Contemporaneously, it is evidently challenging, if not impossible, to overcome the patient’s fear of an examination for a suspected oral lesion (Gao and Guo, 2009; Panzarella et al., 2014; Grafton-Clarke et al., 2019; Thomas et al., 2021).

A dramatic change in patient access to medical facilities and clinical practice is ongoing, in part due to the recent SARS-CoV-2 pandemic. Predictably, patients often develop not only the fear of being affected by malignant lesions but also of exposing themselves to the risk of infection by attending hospitals or doctors’ surgeries. Moreover, the revised day-to-day management of patients and the risk of infection by SARS-CoV-2 requires that fewer patients are seen by medical staff (Campisi et al., 2020; Jacob et al., 2021; Ruiz-Medina et al., 2021; Tulchiner et al., 2021; Popovic et al., 2022; Zubair et al., 2022).

In order to reduce any diagnostic delay, therefore, attention must be paid not only to the clinical-diagnostic skills of professionals but also to their conception of time in the field of medicine, a variable which is often not taken into account in dental practice (Logan and Goss, 2010; Murgod et al., 2011; da Cunha Lima et al., 2021). Time is a crucial, non-recoverable resource which must be fully taken into account when making a diagnosis: a diagnostic delay of only 3 months has been shown to double OSCC measurements with obvious consequences for survival outcomes (Stefanuto et al., 2014). In addition, a delay in treatment is also associated after 20–30 days with statistically significantly reduced survival rates in patients with OSCC (Metzger et al., 2021). This begs the question: which future strategies can be put in place in order to reduce professional delay? There is certainly a need to improve our knowledge regarding OSCC, clinical signs (early and late) and diagnostic strategies in undergraduate and postgraduate training courses. By virtue of ongoing developments in technology (particularly regarding smartphones), there is also a need to increase the value of effective telemedicine practices by means of high-quality systems. Thus, the monitoring of patients most at risk should and must be improved, whether this regards potentially malignant lesions or the risk factors of smoking and alcohol (McCarthy et al., 2021). Similarly, it would be appropriate to include courses which teach the optimization of doctor-patient communication on undergraduate and postgraduate courses in order to effectively manage the communicating of a poor prognosis (or the uncertainty of such a prognosis) (Bazzano et al., 2021).

With regard to the interval between a patient perceiving the symptoms of OSCC and the commencing of diagnostic procedures, the standardization of diagnostic procedures relating to potentially malignant or cancerous lesions is fundamental. Such procedures should expeditiously facilitate the referral of patients from private practices to treatment centers specializing, which are dedicated to OSCC care. Contemporaneously, there is increasing evidence emanating from treatment centers that the development of multidisciplinary teams can facilitate a rapid diagnosis and the prompt treatment of OSCC, thereby improving patient survival and quality of life (Kelly et al., 2013; Licitra et al., 2016; Patil et al., 2016). The presence of a cognitive-behavioral psychotherapist will also be a desirable component of these multidisciplinary teams. Such a figure will be significant regarding the reception and management of anxious patients and the encouraging of appropriate communicative strategies.

The multidisciplinary team could also encourage the creation of patient support groups, including frail patients who could support each other with an exchange of information during shared activities. These patients could, therefore, engage in group and/or recreational activities, the aim of which is to reflect on their health conditions (Bazzano et al., 2021). Finally, the presence of a psychotherapist will be critical in supporting those members of the multidisciplinary team who have the misfortune to suffer from burnout syndrome due to the managing of stressful situations.

## Conclusion

Despite advances in the prevention, diagnostic and therapeutic techniques of OSCC, it remains a disease with a high incidence rate in the population with very low 5-year survival rates, in part due to the fact of diagnostic delay. The patient’s ability to seek help as soon as possible at the initial appearance of signs or symptoms often remains a factor beyond the dentist’s control. There has been in recent years a major change in health care in which there is increasing emphasis directed at the humanization of patient care and the need to devote more time to this end. The authors of this study aspire to encourage their colleagues not to underestimate a central psychological component of delayed care in patients with potentially malignant lesions or oral carcinoma, that is: fear. Moreover, based on a solid and competent interdisciplinary approach with the phycological sciences, further research is required in order to improve our understanding of the role of fear on diagnostic delay and to evaluate different strategies to reduce this delay and improve patient prognosis.

## Author contributions

RM, MB, VP, and GC: conceptualization. RM: methodology. GC: validation and supervision and project administration. RM, MC, and PT: formal analysis. RM, MB, and MC: investigation and data curation. MB and VP: writing—original draft preparation. RM and GC: writing—review and editing. All authors have read and agreed to the published version of the manuscript.

## Funding

RM was supported by Ministero dell’Istruzione, dell’Università e della Ricerca (MIUR) – PON-AIM Line 1 (Id. AIM1892002).

## Conflict of interest

The authors declare that the research was conducted in the absence of any commercial or financial relationships that could be construed as a potential conflict of interest.

## Publisher’s note

All claims expressed in this article are solely those of the authors and do not necessarily represent those of their affiliated organizations, or those of the publisher, the editors and the reviewers. Any product that may be evaluated in this article, or claim that may be made by its manufacturer, is not guaranteed or endorsed by the publisher.
